# Mediators of episodic memory decay across the adult life span

**DOI:** 10.1038/s41598-018-20884-2

**Published:** 2018-02-08

**Authors:** Selene Cansino, Frine Torres-Trejo, Cinthya Estrada-Manilla, Evelia Hernández-Ramos, Joyce Graciela Martínez-Galindo, Tania Gómez-Fernández, Mariana Ayala-Hernández, María Dolores Ramírez-González, Silvia Ruiz-Velasco

**Affiliations:** 1 Laboratory of NeuroCognition, Faculty of Psychology, National Autonomous University of Mexico, Mexico City, 04510 Mexico; 2 Department of Pharmacology, School of Medicine, National Autonomous University of Mexico, Mexico City, 04510 Mexico; 3 Applied Mathematics and Systems Research Institute, National Autonomous University of Mexico, Mexico City, 04510 Mexico

## Abstract

The ability to remember the details of our own experiences declines gradually as we get old. The reason for this decay has been attributed to several factors besides age, such as education, nutrient intake and health status. However, the influence of these factors has mainly been examined individually and rarely together. Here we identify those factors that jointly act as mediators of episodic memory decay across the adult life span. We examined source memory in a lifespan sample of 1557 healthy adults. A total of 70 physical, biological and lifestyle variables were measured and introduced into a structural equation model as potential mediators that intervene between age and source memory. Only 14 mediator variables reliably mediated source memory decay; notably, eight of these variables have an effect on the cardiovascular system. The model unequivocally highlights that the mediators that may impair cardiovascular functioning also impact brain resources involved in episodic memory. We identified the factors that are relevant to episodic memory decline when they interact together as occurs in real life.

## Introduction

One of the most worrying problems people experience as they age is an increase in memory failures. For instance, the ability to recollect the details that surrounded our personal living experiences decreases gradually with age. The memory for our own experiences is called episodic memory^1^, and it is encoded as representations that include the event itself together with the context in which it occurred, such as spatial and timing information and thoughts or emotions present at the moment. It is precisely the contextual information that becomes less accessible and more inaccurate as we get old. In an experimental setting, the ability to retrieve the context or source information related to a previous experience can be achieved by asking participants to report the context in which each event occurred in a previous encoding session. This procedure objectively measures the ability to remember the elements that are more vulnerable to being lost from our episodic representations. Empirical evidence^2^ based on this procedure showed that source memory for spatial information decays linearly at a rate of −0.6% per year across the adult life span.

The identification of factors that mitigate or intensify cognitive decay has been a research topic investigated for three decades. According to a meta-analytic study^3^, one factor consistently shown to influence cognition is physical activity; however, its effects appear to be selective, with particular benefit for executive-control processes that depend on prefrontal cortices. In addition, several nutrients have been identified as relevant for preventing cognitive impairment. For example, low consumption of fish, an important source of omega-3 fatty acid, has been associated with cognitive impairment^4^. Likewise, low levels of antioxidants such as vitamin E in serum have been associated with poor memory^5^. Regular engagement in mental and social activities has also been correlated with increased memory performance^6^.

Although several factors have been identified that influence episodic memory in particular or cognition in general, their effects have been mainly analyzed individually^7,8^ and rarely together in the same sample. These previous studies focused mostly on older adults^9,10^, or the factors were examined using univariate analysis^11^. However, in everyday life, we are simultaneously exposed to several of these factors, and it is unknown whether they would still have an effect on episodic memory when they interact. Therefore, to test which of these factors remains influential in episodic memory in the presence of other factors, we examined 120 variables in a healthy adult lifespan sample comprised of 1557 individuals between 21 and 80 years old; age and sex were equally distributed across the age range (Supplementary Table S1). The current study makes a unique contribution as the first one that includes almost all variables that have been empirically documented to be predictors of memory or age-related cognitive decline. Moreover, by assessing these factors in an adult lifespan sample, we were able to identify predictors of source memory performance that are relevant across adulthood and not only for an exclusive age group. We used structural equation modeling (SEM) to identify the most plausible model that includes only reliable mediators of the effects of age on source memory; all mediators were observed variables to allow their direct comparison with everyday measurements of these variables and with other studies. The factors measured (Supplementary Table S2) included demographic variables, diseases with which participants have been diagnosed, biological, physical and physiological measures, nutrient consumption, medication intake, tobacco, drugs and alcohol consumption, lifestyle variables such as physical, mental, social and cultural activities, experience of stressful events, metamemory (participants’ affects and beliefs about their own memory), depression and general intellectual and cognitive state.

## Results

**Figure 1:**
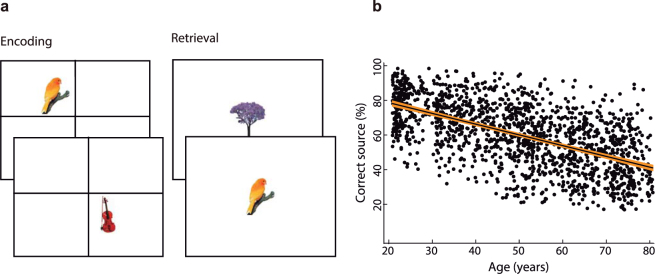
(**a**) Examples of the images used in the source memory paradigm. During encoding, the display was divided into quadrants, and the stimuli were presented randomly in one of the quadrants, whereas during retrieval, they were presented at the center of the display. (**b**) Source accuracy in a life span sample of 1557 adults between 21 and 80 years of age as a function of participant age. The solid line represents linear regression fit, and the orange band represents the 95% confidence interval.

We measured the ability to retrieve the spatial context (quadrant in the screen) in which images were presented during an encoding phase in all participants (Fig. 1a). In this phase, participants classified each image as natural or artificial. In the following retrieval phase, new images and previously presented images were displayed at the center of the screen. The participants’ task was to indicate the quadrant in which the image was originally presented or if the image was new. This five-choice procedure was achieved using a five-key response panel, which had four keys distributed as the screen’s quadrants (see supplementary materials for more task details). Source memory accuracy as a function of age in the adult life span sample is displayed in Fig. 1b.

**Figure 2:**
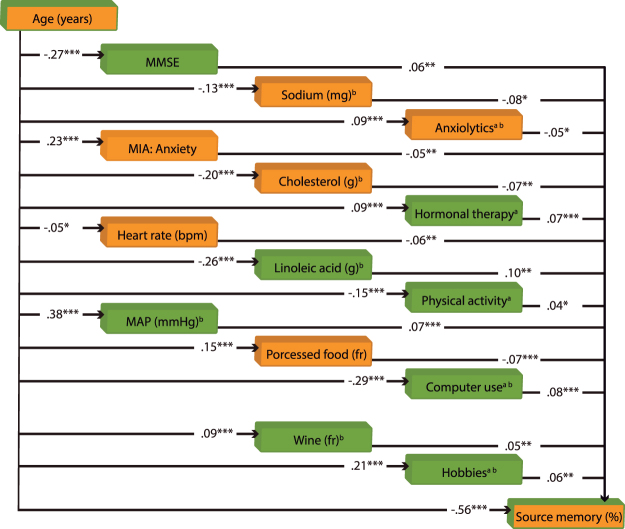
Structural equation model comprising only variables that significantly mediated the effects of age on episodic memory decline across the adult lifespan. Path coefficients are standardized estimates. Positive predictors of source memory performance are presented in green squares, and negative predictors in orange squares. MMSE = Mini Mental State Scale, MIA = Metamemory in Adulthood Scale, MAP = mean arterial pressure, fr = frequency. ^a^Total intake or time = frequency × duration. ^b^Log transformed variable. *P < 0.05, **P < 0.01, ***P < 0.001.

First, SEM was used to test an a priori hypothetical model comprising variables that were considered potential mediators of the effects of age on source memory. This procedure has the power to identify influential factors within a larger pool of variables because it accounts for all possible relationships among the variables included in the model. However, a limitation of SEM is that it can only indicate whether the model fitted and corroborated by the data is plausible; it does not imply that the model is correct or true. Participant age was the only exogenous variable, and source memory accuracy the only outcome variable. Linear regression analyses (Supplementary Table S3) were conducted between all variables and source accuracy to select only those variables that had a significant (*P* < 0.05) effect on source memory. Seventy variables turned out to be significant and were included in the first SEM analysis as potential mediators. After the model was fitted, only the paths of 20 endogenous variables had significant *t* values (*P* < 0.05), thus the model was fine-tuned by eliminating the mediator variables that had paths that were no longer significant. The SEM estimation was repeated, and results revealed that the paths for six mediator variables were no longer significant. Therefore, they were eliminated from the model. The SEM analyses conducted with 14 mediator variables revealed that all path coefficients in the model were significant (Fig. 2). A path coefficient indicates the direct effect of a variable assumed to be causal on another variable assumed to be an effect. The correlation matrix of this model is presented in Supplementary Table S4, and the SEM results are depicted in Supplementary Table S5. The goodness-of-fit for this final model was *χ*^2^ (85) = 243.56, *P* < 0.0001; relative *χ*^2^ = 2.87; comparative fit index (CFI) = 0.96; root mean square error of approximation (RMSEA) = 0.035; and standardized root mean squared residual (SRMS) = 0.032.

**Figure 3:**
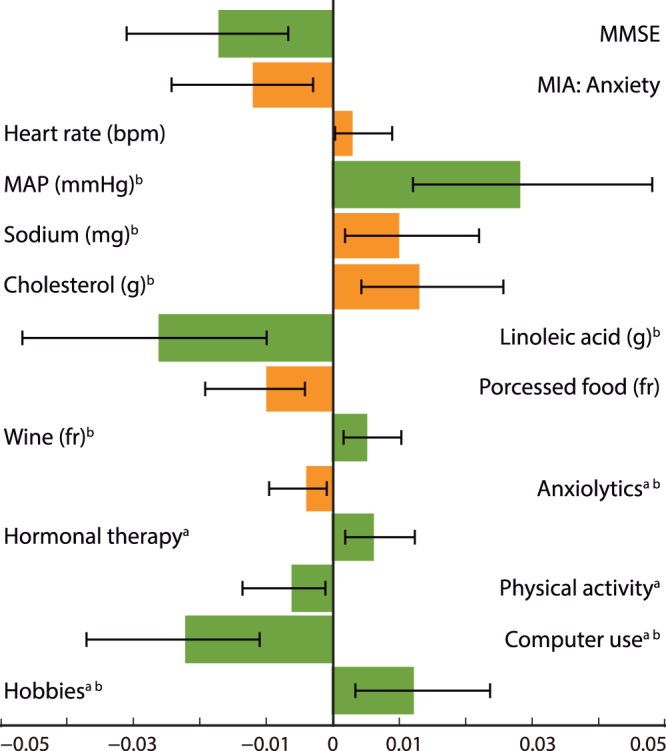
Estimates of the indirect effects (β) for each of the mediator variables and 95% bias- corrected confidence intervals calculated using a bootstrap procedure with 20,000 samples. Indirect effects were significant for all mediator variables. However, note that some positive predictors (green) and negative predictors (orange) had opposite indirect effects when mediating the effects of age on source memory. MMSE = Mini Mental State Scale, MIA = Metamemory in Adulthood Scale, MAP = mean arterial pressure, fr = frequency. ^a^Total intake or time = frequency × duration. ^b^Log transformed variable.

The CFI compares the model of interest with the null or independence model, in which variables are assumed to be uncorrelated. The RMSEA is a noncentrality-based index, i.e., it does not test whether the null hypothesis is true. This is in contrast to *χ*^2^, which tests the hypothesis that *χ*^2^ = 0, the “central” chi-square distribution. RMSEA provides a mechanism to adjust for sample size, and the goal is for the population data to closely fit the model. The SRMS is an absolute fit index because it does not use an alternative model as a basis for comparison; rather, this index examines the discrepancy between the sample covariance matrix and the model covariance matrix. The coefficient of determination (CD) of the model was 0.53; this denotes the proportion of dependent variable variation that is predicted by the independent variables. The statistical powers for each of the three models fitted were calculated by testing the covariance structure model using RMSEA^12,13^. Setting the alpha level to 0.05, the null RMSEA to 0.05 and the alternative RMSEA to 0.08, we found that the three models each had a statistical power greater than 0.99. The indirect effects of each mediator variable were significant as revealed by bootstrapping (Fig. 3). However, the total indirect effect of age through all mediator variables was not significant [β (95% CI) = −0.023 (−0.057, 0.013)]. Because the direct effect of age on source memory was β = −0.595, the proportion of the total effect mediated was 0.037.

## Discussion

The final model shows that only fourteen variables reliably mediated the effects of age on source memory from a total of 70 potential mediators. This outcome reveals that when we are exposed to several factors, as occurs in real life, only few of them actually have an effect on episodic memory decline, specifically those that are sufficiently powerful to influence episodic memory even in the presence of other competitive factors.

Predictors from several domains remained in the model. However, eight of them may be conceived of as closely related: heart rate, mean arterial pressure (MAP), sodium, cholesterol, linoleic acid, wine and processed food intake, and physical activity. Notably, all these variables have an effect, exclusive or not, on the cardiovascular system. Heart rate decreases with advancing age^14^, while MAP tends to increase^15^, as was observed here (Fig. 2). Their effects on source memory accuracy can be explain by the fact that heart rate deceleration occurs when attention is focused on the external environment to enhance stimulus receptivity^16^ and encoding, whereas MAP, i.e., the increase of blood perfusion pressure delivery to the organs, in particular to the brain, enhances cognition as revealed by cerebral blood flow measurements^17^. The intake of sodium and cholesterol diminishes with age, but that for processed food increases, probably due to several conditions that cause malnutrition in older individuals^18^. The consumption of these nutriments is negatively associated with source memory due to their effect on the cardiovascular system, which in turn affects cerebrovascular function. Elevated sodium intake increases blood pressure, which is associated with several brain dysfunctions^19^. High cholesterol intake may be associated with carotid atherosclerosis, which interferes with cerebral blood flow^20^. Processed food is the major source of trans-fatty acid consumption, which increases plasma concentrations of low-density-lipoprotein cholesterol^21^.

An outstanding finding was that linoleic acid (omega-6) intake was associated with higher source memory performance instead of the alpha-linoleic acid (omega-3), which has been typically related to cognitive functions. However, this outcome is not surprising because both essential fatty acids participate in neural signaling transduction mechanisms as lipid secondary messengers that modulate several functions, such as neurotransmitter release^22^. Additionally, these polyunsaturated fatty acids preclude cardiovascular dysfunction by lowering serum levels of low-density-lipoprotein^23,24^. Cell membrane fatty acid composition depends on the intake of these fatty acids through the diet, though their benefits depends on an intake ratio of 1:1 of omega-6:omega-3 as revealed by anthropological, epidemiological and molecular studies^24^. The positive association between wine consumption and source memory accuracy essentially should be attributed to its flavonoid compounds, which have powerful antioxidant and anti-inflammatory properties that contribute to vascular endothelium repair of damage caused by reactive oxygen species^25^. Likewise, the alcohol component of wine, if its consumption is low to moderate, has protective effects on the cardiovascular system by suppressing low-density-lipoprotein oxidation. However, due to a lack of consensus, the level of alcohol consumption considered moderate is ambiguous and may be miscalculated. Of note, a meta-analysis indicated that the association between alcohol consumption and cardiovascular diseases showed a U-shaped curve, whereby abstainers and heavy drinkers had a higher risk of stroke than light to moderate drinkers^26^. Physical activity enhances oxygen uptake, a prominent precursor of brain vasculature increment, which indirectly and directly benefits cognition, such as through increased gray matter volume in the prefrontal cortex and hippocampus, which are regions relevant to episodic memory^27^.

The detrimental effect of anxiolytic intake on source memory was foreseen because several studies have confirmed that benzodiazepines, the most common anxiolytic drug prescribed, impair episodic memory. Their effects are dose-dependent and increase with advancing age due to the decline in the ability to metabolize and eliminate drugs^28^. The benzodiazepines stimulate the gamma-amino butyric acid (GABA)-A receptor, reducing the excitability of neurons in the cortex and limbic system. Although the research on the effects of hormonal therapy on cognition has encountered conflicting results, here, we observed that hormonal therapy enhanced source memory performance. Estrogens influence cholinergic neurons in the basal forebrain, which is the main producer of acetylcholine that is released in the hippocampus and cerebral cortex^29^. The neurotransmitter and neuromodulator acetylcholine is implicated in memory and learning. Moreover, estrogens also directly act on hippocampal structure and function.

The time spent on the computer and engaged in hobbies were the only mental activities that had an effect on episodic memory, indicating that only activities that demand cognitive effort and interactive coordination among different systems, such as motor and sensory, had an effect on cognition^30^. As in animal studies housed in enriched environments^31^, mentally challenging activities lead to functional and structural brain changes in humans^32^ that may support memory enhancement. Anxiety related to memory performance increases with advancing age, and this perception has negative effects on source memory accuracy. The experience of anxiety influences memory performance because other processes interfere by demanding attention or resources destined for memory representations^33^.

The Mini-Mental State Exam (MMSE) measures cognitive impairment; in the current study, none of the participants showed cognitive impairment according to our inclusion criteria (score > 24, which is considered normal cognition^34,35^. Therefore, even subtle differences in normal cognitive function influenced source memory performance, indicating that the ability to recollect contextual information benefits from full efficiency in all cognitive domains. Although some authors^36^ have proposed using higher scores to ensure the absence of cognitive impairment, scores between 24 and 27 could be considered small failures due to normal cognitive decline associated with aging. Moreover, each participant was able to understand and perform the highly complex memory tasks, suggesting that all participants were cognitive healthy. However, we cannot discard the possibility that some participants were affected by cognitive impairment because the MMSE has a false negative rate of 5%^37^.

The indirect effects of each mediator were low but significant in comparison with the direct effect of age on this type of memory. However, the collective effect of all mediators did not significantly account for the effects of age on source memory accuracy. This outcome is not surprising when multiple mediators are tested simultaneously because the total indirect effect results from the sum of all indirect effects, and not all mediators affected source memory accuracy in the same direction. In the final model, mediators with opposing indirect effects offset each other, leading to an insignificant total indirect effect. Nevertheless, this outcome does not undermine the conclusion that individual mediators had a significant indirect effect on source memory.

One limitation of the current study is that our results are not representative of the population because the sample was selected by non-probabilistic methods; this procedure was adopted because the objective of the study was to identify factors that mediate the relationship between age and source memory rather than to make an inference from the population. Another limitation of the study is that some of our variables may be affected by random measurement error because they were assessed through participants’ reports, which relies on participants’ accuracy and memory. However, this method was necessary to obtain information on a wide range of variables.

The model provides useful information on which factors really have enough power to affect episodic memory when they jointly interact as they do in real life. One finding the model reveals is the connection between brain function and the cardiovascular system. Most of the factors (heart rate, mean arterial pressure, sodium, cholesterol, linoleic acid, wine and processed food intake, and physical activity) in the model likely mediate the effects of age on source memory accuracy through their effects on the cardiovascular system, which in turn positively or negatively impacts brain resources; however, some of these factors are also known have direct effects on brain function. Two factors (anxiolytic intake and hormonal therapy) impact episodic memory by acting directly on brain function through their pharmacological effects. The identification of other factors, such as computer use and hobbies, demonstrates that more demanding mental activities positively mediate the effects of age on source memory performance. In addition, cognitive integrity (MMSE) and the absence of anxiety during memory functions positively mediate the decay of spatial source memory. Remarkably, the mediators included in the model collectively influence brain function through diverse biological, physical and cognitive mechanisms, as is expected in everyday life. This study also shows that factors that have negative effects on episodic memory may be easy to avoid, and those that have positive effects remain a possible choice to preserve memory, the most important human cognitive ability.

## Methods

### Participants

A total of 1557 healthy adults between the ages of 21 and 80 participated in this study (784 women and 773 men). Approximately the same number of men and women were distributed among the six decades in the age range (Supplementary Table S1). The complete sample included 1657 participants; 100 individuals were excluded because their data from the episodic memory task were lost due to technical difficulties. Participants were recruited in Mexico City through advertisements, appeals to community groups, flyers, and word of mouth. To be eligible for the study, participants had to have at least eight years of education, not have been addicted to drugs or alcohol, not have taken any medication that acted on the nervous system for the previous six months, not have neurological or psychiatric diseases, not have experienced head trauma and have normal or corrected-to-normal vision. Additionally, participants were required to obtain specific performance scores on psychological tests to ensure that they were not suffering from depression, dementia, or intellectual difficulties. The performance required was a score ≤ 20 on the Beck Depression Inventory (BDI), a score ≥ 24 on the Mini-Mental State Exam (MMSE), and a score ≥ 26 on the vocabulary subtest of the Wechsler Adult Intelligence Scale-Revised (WAIS-R). Supplementary Table S1 displays the participants’ scores on these tests by decade. All participants provided informed consent and received a monetary reward for his/her participation. The study was approved by the Bioethics Committee of the School of Medicine at the National Autonomous University of Mexico. All experiments were performed in accordance with relevant guidelines and regulations.

### Measures

Metamemory in Adulthood^38^ is a scale composed of 108 questions and statements measuring knowledge, affects and beliefs about our own memory. A 5-point Likert scale was used to assess the degree of agreement with various sentences or the frequency of some behaviors. The scale measures seven dimensions: use of memory strategies, knowledge of memory tasks, knowledge of one’s own memory capacities, perception of memory change, relationship between anxiety and memory performance, achievement on memory tasks, and locus of control in memory abilities. The scale was translated into Spanish, and then the translated version was reviewed independently by 10 judges for linguistic and cultural validation consisting of assessing the equivalence of concepts in the questionnaire and adapting the concepts to the Spanish culture.

Food Frequency Questionnaire^39^ is a semi-quantitative instrument composed of 116 food items designed to assess dietary intake. Frequency of consumption of each food item over the previous year is measured using 10 frequency categories ranging from never, less than once a month, 1–3 times per month, once a week, 2–4 times per week, 5–6 times per week, once a day, 2–3 times per day, 4–5 times per day and 6 times per day. Nutrient intake was estimated using the Evaluation System of Nutritional Habits and Nutrient Consumption (SNUT) software developed by the National Institute of Public Health^40^. The validity and reproducibility of the Food Frequency Questionnaire have been confirmed for individuals living in Mexico City (e.g.,^39^). In addition, we asked participants how often they consumed canned food and processed food using the same frequency scale.

Social Readjustment Rating Scale (SRRS)^41^ is comprised of 43 positive and negative stressful life events. Participants are requested to respond if they had experienced each of the events in the last 12 months. The scale estimates the magnitude of readjustment to accommodate to a life event. The ratings assigned to each event were obtained from a sample of 394 persons that estimated the amount of social readjustment required for each event. Because the rates assigned to each event have been challenged, we scored the scale as the number of stressful events endorsed by each participant.

Lifestyle Questionnaire was created on purpose for the current study to examine education, occupation, income, health status, medication intake, tobacco, drug and alcohol consumption, and cultural, social, mental and physical activities. The questionnaire was applied as a semi-structured interview by psychologists who were carefully trained before collaborating in the study. Income was classified into nine categories ranging from less than 1000 Mexican pesos per month to between 1000–2000, 2000–4000, 4000–7000, 7000–10000, 10000–15000, 15000–20000, 20000–30000 or 30000 or more Mexican pesos per month. The retirement variable measured the time elapsed between retirement and the interview date. Health status was examined for the following systems: nervous, respiratory, cardiovascular, immune, sensory, digestive, renal, reproductive, endocrine, musculoskeletal, hepatic portal, and sleep. Participants were requested to report the diseases they have had formally diagnosed by a physician in each of the above systems during their lifetime.

Medications for antidepressants, neuroleptics, nootropics, hypnotics, anxiolytics, analgesics, amphetamines and hormonal therapy were assessed. The consumption of pharmaceutical drugs from each category was evaluated by asking participants if they had taken the medicine for the typical purpose or by asking for the reasons they are usually prescribed; the classification names listed above were not used in the interview. Participants were asked to provide the medication name, age of initial consumption and intake frequency and duration. The medicines were categorized afterwards by pharmaceutical specialists.

We investigated consumption of drugs (cannabis, hallucinogens, and cocaine), tobacco and alcohol by asking participants to communicate age of onset of consumption, frequency and duration of consumption and time since last consuming the drug. Additionally, for tobacco and alcohol, participants indicated the number of cigarettes and glasses of alcohol they usually have when they smoke and drink. Likewise, participants reported with which frequency they drink different alcoholic beverages (beer, wine, liqueur and spirits). Frequency was classified into 10 categories (never, once a year, three times per year, six times per year, once per month, two or three times per month, one or two times per week, three or four times per week, almost every day and daily). The amount of alcohol consumption was calculated as total grams per week based on the following equivalences: 6 gr of alcohol/200 mL beer, 9.6 gr of alcohol/100 mL wine, 25 gr of alcohol/50 mL liqueur, and 42 gr of alcohol/50 mL spirit.

Participants were requested to report the frequency and time they spent in physical activity (aerobic and anaerobic exercise), mental activity (watching television, listening to the radio, using the computer and reading), attending cultural events (film screenings, theater plays, exhibitions, concerts, conferences or courses), attending social events (parties or reunions) and hobbies. Participants were asked for the type of exercise they performed most frequently. Additionally, participants indicated the genres of television, radio and literature they most often chose. Likewise, the kind of activity they most frequently performed on the computer was assessed. The same 10 frequency categories described above for drug intake were used for these variables.

The scores obtained on the BDI, MMSE and WAIS-R Vocabulary subtest screening tests were also included in the structural equation modeling analyses.

### Stimuli

A total of 110 color images representing natural and artificial common objects (50% from each category) were used in the source memory paradigm, and 2 of them were presented at the beginning of the encoding and retrieval tasks and were not analyzed (Fig. 1a). Additionally, another 12 images were employed in a practice session. From the set of 108 images, 72 of them were randomly selected (equal proportion of natural and artificial objects) for each participant to be presented during the encoding phase, and the complete set of 108 images was randomly presented at the retrieval phase. Each image subtended vertical and horizontal visual angles ranging from 2.9° to 4.3° and were displayed on a white background screen.

### Procedure

The study started in 2003 and lasted six and a half years. Approximately the same number of participants from each decade in the age range was evaluated each year. Participants attended two sessions of about two hours each. Graduate psychologist collaborators conducted the experimental sessions and applied the instruments after several months of training. The training consisted of learning how to interview, apply psychological tests, evaluate visual acuity, and administer the memory tasks. To ensure that the collaborators had acquired the necessary skills before being allowed to formally carry out the study, they were observed several times and evaluated through a Gesell chamber while conducting interviews and applying psychological tests. The collaborators were also supervised and evaluated while they administered the memory tasks to guarantee that the procedure was applied consistently to all of the participants. Potential participants were checked through prescreening questions if they fulfilled the inclusion and exclusion criteria prior to being invited to attend the first session. The first session occurred in a silent room in which only the participant and the experimenter were present. At the beginning of this session, participants were further interviewed to confirm that they satisfied the inclusion criteria. Afterwards, participants were tested with the WAIS-R Vocabulary subtest, the MMSE and the BDI, and their vision was tested. Participants that were eligible for the study were asked to provide their informed consent. Afterwards, participants completed the Lifestyle Questionnaire, followed by the Food Frequency Questionnaire and the SRRS, which were completed in a counterbalanced order. Then, the Annett Hand Preference Questionnaire was administered. At the end of the session, participants had the *Metamemory in Adulthood* questionnaire explained, which was then handed to them to be answered at home. Participants were also asked to record all foods they ate for three days in special formats (data not analyzed here). Finally, participants’ weight and height were measured.

In the second session, participants’ glucose, cholesterol and triglycerides were measured in a non-fasting state with the Accutrend Plus System, Roche Diagnostics, Rotkreuz, Switzerland. These measurements were taken in a counterbalanced order. Afterwards, participants performed a working memory task (data not shown) in addition to the source memory task, which consisted of an encoding and a retrieval phase, in a sound-dampened chamber. Only one room was used for this session. The participants were seated in a high-back armchair 100 cm away from the monitor screen. Blood pressure and heart rate were measured with a digital upper arm sphygmomanometer Hem-712C, Omron, Kyoto, Japan. Mean arterial pressure (MAP) was estimated as [(2 × diastolic blood pressure) + systolic blood pressure]/3. Skin conductance responses were recorded by placing an electrode in the annular and medium fingers of the non-dominant hand (data not presented here). The response panel, adapted for right- or left-handed participants, was located on a platform on the left or right armchair according to the participant’s handedness. The response panel consisted of four keys arranged in two columns of two rows each to be pressed by the index and middle finger, and a fifth key located in the lower portion of the response panel to be pressed by the thumb. The four keys represented the quadrants of the screen where the images were presented during the encoding phase. Only the two keys in the second row were used during the encoding phase, while all five keys were used during the retrieval phase. The participants performed a training session that involved a shorter version of the encoding and retrieval tasks to learn how to use the response panel. The stimulus presentation and response recording were controlled by E-Prime software v1.0, Psychological Software Tools, Pittsburgh, PA, USA.

### Source memory paradigm

Throughout the encoding task, a cross divided the screen into quadrants; the center of the cross was in the middle of the screen. The images were randomly displayed in one of the quadrants with the same probability of appearing in each of them. The images were displayed at a distance ranging from 0.5° to 1.25° away from the axes of the cross dividing the screen. Each trial started with the presentation of an image for 1000 ms followed by a 3000 ms period when only the cross remained on the screen. The task consisted of classifying whether the images represented a natural or an artificial object by pressing one of two keys. Participants were able to respond after the onset of the stimulus during a period of 3500 ms. Participants were instructed to concentrate on the encoding task because they knew that their memory would be tested.

Approximately three minutes after the encoding task was completed, the retrieval task began. The screen was not divided into quadrants, and the images were presented in the center of the screen. The same timing used in the encoding task was used in the retrieval task. Participants were asked to judge whether the image was new or old. If the image was old, they indicated the quadrant where the image was originally presented during the encoding phase by pressing one of the four keys that represented each quadrant of the screen. If the image was new, participants used their thumb to press the lower (fifth) key on the response panel. Participants were instructed to randomly select one of the four quadrants if they were confident that the image was old but were unable to remember its exact position.

### Data analysis

Structural equation modeling (SEM) was conducted by using Stata v. 13 Texas, USA with the maximum likelihood estimation procedure. A hypothetical model was developed a priori based on previous empirical findings that demonstrated that the variables included in the model were associated with or had an effect on episodic memory, memory in general or cognition. The model consisted of one exogenous variable: participant’s age. The ultimate outcome variable was source memory accuracy, which was estimated as the percent of recognition hits accompanied by a correct source response. Note that the images that were incorrectly classified during the encoding task were excluded from the analyses. The rest of the variables were considered endogenous mediator variables that intervene between age and source memory in a causal sequence. Thus, we estimated the magnitude of the direct effect of age on source memory not mediated by other variables in the model. Also, we calculated the indirect effect of age mediated by the other variables in the model, and the indirect effect of each mediator variable by multiplying the coefficients for the two linked direct effects. Data were not developed by any reduction technique. Therefore, only observed variables were included in the model because they represent precise evidence of the domains of interest, and they can be directly compared with predictors from other studies.

A total of 120 variables were considered as potential mediator variables, and none of them had missing values. Descriptive analyses were conducted to assess all study variables. Those variables with skewness exceeding ±3 were natural log-transformed. Then, we applied linear regression analyses between all variables and the correct source to select the variables that would be entered in the SEM analyses. Only those variables with a significant (*P* < 0.05) effect on source memory were selected. Linear regressions were not corrected for multiple comparisons because they were not used to test alternative hypotheses. The development of the model was completed in several steps. First, all the variables that had a significant effect on source memory accuracy were included in the SEM analysis. After the paths were fitted, the model was fine-tuned by eliminating endogenous variables, which paths had *t* values that were no longer significant (*P* > 0.05). Then, we repeated the SEM analysis with only the endogenous variables that remained significant. If necessary, we repeated this procedure until all mediator variables’ paths were significant in the model. Goodness-of-fit was assessed with the *χ*^2^ likelihood ratio with degrees of freedom, relative or normed *χ*^2^ (*χ*^2^/degrees of freedom), the comparative fit index (CFI), the root mean square error of approximation (RMSEA) and the standardized root mean squared residual (SRMS). Finally, we performed a bootstrapped test of mediation^42^ with 20,000 replications to estimate the 95% bias-corrected confidence intervals for each mediator variable and the total indirect effect.

### Data availability

The data that support the findings of this study are available from the corresponding author upon reasonable request.

## Electronic supplementary material

Supplementary information

## References

[1] Tulving, E. In Organization of Memory (eds Tulving, E. & Donaldson, W.) 381–403 (Academic Press, New York, 1972).

[2] Cansino S (2013). The rate of source memory decline across the adult life span. Dev. Psychol..

[3] Colcombe S, Kramer AF (2003). Fitness effects on the cognitive function of older adults: a meta-analytic study. Psychol. Sci..

[4] Kalmijn S, Feskens EJ, Launer LJ, Kromhout D (1997). Polyunsaturated fatty acids, antioxidants, and cognitive function in very old men. Am. J. Epidemiol..

[5] Perkins A (1999). Association of antioxidants with memory in a multiethnic elderly sample using the Third National Health and Nutrition Examination Survey. Am. J. Epidemiol..

[6] Small BJ, Dixon RA, McArdle JJ, Grimm KJ (2012). Do changes in lifestyle engagement moderate cognitive decline in normal aging? Evidence from the Victoria Longitudinal Study. Neuropsychol..

[7] Hultsch DF, Hammer M, Small BJ (1993). Age differences in cognitive performance in later life: Relationships to self-reported health and activity life style. J. Gerontol..

[8] Santos NC (2014). Clinical, physical and lifestyle variables and relationship with cognition and mood in aging: Across-sectional analysis of distinct educational groups. Front. Aging Neurosci..

[9] Albert MS (1995). Predictors of cognitive change in older persons: MacArthur studies of successful aging. Psychol. Aging.

[10] Hansson JA, Hagberg B (2005). Determinant factors contributing to variations in memory performance in centenarians. Int. J. Aging Hum. Dev..

[11] Nilsson L-G (1997). The Betula prospective cohort study: Memory, health, and aging. Aging Neuropsychol. C..

[12] MacCallum RC, Browne MW, Sugawara HM (1996). Power analysis and determination of sample size for covariance structure modeling. Psychol. Methods.

[13] Preacher, K. J. & Coffman, D. L. Computing power and minimum sample size for RMSEA [Computer software]. Available from http://quantpsy.org/ (2006).

[14] Kostis JB (1982). The effect of age on heart rate in subjects free of heart disease. Studies by ambulatory electrocardiography and maximal exercise stress test. Circulation.

[15] Finkel D (2003). Genetic and environmental influences on decline in biobehavioral markers of aging. Behav. Genet..

[16] Lacey, J. L. Somatic response patterning and stress: Some revisions of activation theory in Psychological Stress: Issues in research (eds Appley, M. H. & Trumbull, R.) 14–42 (Appleton-Century-Crofts, New York, 1967).

[17] Popa-Wagner A, Buga AM, Popescu B, Muresanu D (2013). Vascular cognitive impairment, dementia, aging and energy demand. A vicious cycle. J. Neural. Transm..

[18] Hickson M (2006). Malnutrition and ageing. Postgrad. Med. J..

[19] Manolio TA, Olson J, Longstreth WT (2003). Hypertension and cognitive function: Pathophysiologic effects of hypertension on the brain. Curr. Hypertens. Rep..

[20] Schreurs BG (2010). The effects of cholesterol on learning and memory. Neurosci. Biobehav. Rev..

[21] Ascherio A, Willett WC (1997). Health effects of trans fatty acids. Am. J. Clin. Nutr..

[22] Haag M (2003). Essential fatty acids and the brain. Can J Psychiatry.

[23] Mangal, D., Uboh, C. E. & Soma, L. R. In Arachidonic Acid Dietary Sources and General Functions (eds Dumancas, G. G. Murdianti, B. S. & Lucas, E. A.) 33–49 (Nova Science Publishers, New York, 2013).

[24] Simopoulos AP (2006). Biomed evolutionary aspects of diet, the omega-6/omega-3 ratio and genetic variation: Nutritional implications for chronic diseases. Biomed. Pharmacother..

[25] Markoski MM, Garavaglia J, Oliveira A, Olivaes J, Marcadenti A (2016). Molecular properties of red wine compounds and cardiometabolic benefits. Nutr. Metab. Insights.

[26] Reynolds K (2003). Alcohol consumption and risk of stroke: a meta-analysis. JAMA.

[27] Miller DI, Taler V, Davidson PSR, Messiera C (2012). Measuring the impact of exercise on cognitive aging: Methodological issues. Neurobiol. Aging.

[28] Griffin CE, Kaye AM, Bueno FR, Kaye AD (2013). Benzodiazepine pharmacology and central nervous system-mediated effects. Ochsner J..

[29] Gibbs RB (2010). Estrogen therapy and cognition: A review of the cholinergic hypothesis. Endocr. Rev..

[30] Wang, H. X., Xu, W. & Pei, J. J. Leisure activities, cognition and dementia. Biochim. Biophys. Acta **1822**, 482–491 (2012). 10.1016/j.bbadis.2011.09.002 21930203

[31] Black JE, Greenough WT, Anderson BJ, Isaacs KR (1987). Environment and the aging brain. Can. J. Psychology.

[32] Boyke J, Driemeyer J, Gaser C, Buechel C, May A (2008). Training-induced brain structure changes in the elderly. J. Neurosci..

[33] Moran TP (2016). Anxiety and working memory capacity: A meta-analysis and narrative review. Psychol. Bull..

[34] Folstein MF, Folstein SE, McHugh PR (1975). Mini-mental state. A practical method for grading the cognitive state of patients for the clinician. J. Psychiatr. Res..

[35] Mungas D (1991). In-office mental status testing: a practical guide. Geriatrics.

[36] Perneczky R (2006). Mapping scores onto stages: mini-mental state examination and clinical dementia rating. Am. J. Geriatr. Psychiatry.

[37] Anthony JC, LeResche L, Niaz U, von Korff MR, Folstein MF (1982). Limits of the ‘Mini-Mental State’ as a screening test for dementia and delirium among hospital patients. .Psychol. Med..

[38] Dixon RA, Hultsch DF, Hertzog C (1988). The Metamemory in Adulthood (MIA) questionnaire. Psychopharmacol. Bull..

[39] Hernández-Ávila M (1998). Validity and reproducibility of a food frequency questionnaire to assess dietary intake of women living in Mexico City. Salud Pública México.

[40] Hernández-Ávila, J. E., González-Aviles, L. & Rosales-Mendoza, E. Manual de usuario. SNUT Sistema de evaluación de hábitos nutricionales y consumo de nutrimentos (Instituto Nacional de Salud Pública, Cuernavaca, México, 2000).

[41] Holmes TH, Rahe RH (1967). The social readjustment rating scale. J. Psychosom. Res..

[42] Preacher KJ, Hayes AF (2008). Asymptotic and resampling strategies for assessing and comparing indirect effects in multiple mediator models. Behav. Res. Methods.

