# Supplementary material for: Mediators of episodic memory decay across the adult life span
Source: Sci Rep. 2018 Feb 8;8:2610. doi: 10.1038/s41598-018-20884-2 (PMC5805709; doi:10.1038/s41598-018-20884-2)
Supplement: Supplementary file 1 — Supplementary information [file 41598_2018_20884_MOESM1_ESM.pdf]

SUPPLEMENTARY INFORMATION

Mediators of episodic memory decay across the adult life span

Selene Cansino*, Frine Torres-Trejo, Cinthya Estrada-Manilla, Evelia Hernández-Ramos, Joyce Graciela Martínez-Galindo, Tania Gómez-Fernández, Mariana Ayala-Hernández, María Dolores Ramírez-González and Silvia Ruiz-Velasco

Full results. Supplementary Table S2 displays the descriptive analyses conducted on all variables. Because skewness increased after been log-transformed for some variables, no transformed data were used for these variables. The subsequent analyses were also conducted with data without any transformation, and results revealed no differences from those performed with transformed data. The results of the linear regression analyses computed between all variables and correct source accuracy are displayed in Supplementary Table S3. A total of 70 linear regressions turned out to be significant, thus these variables were included as mediator variables in the first SEM analyses. The fitted model was fine-tuned by eliminating mediator variables with paths that were no longer significant; only 20 mediator variables were retained in the model. The second SEM estimation computed with these variables revealed that the paths for six other mediator variables were not significant, thus, they were eliminated from the model. The SEM analysis was conducted again with only 14 mediator variables. All paths in this model were significant (Figure 2).

The correlation matrix of the final model is presented in Supplementary Table S4. The inspection of modification indices showed some model misspecifications due to related

variables. One of them was observed between the scores from the MMSE and the Anxiety scale of the MIA. Another misspecification was observed between heart rate and mean arterial pressure, and the others were observed between the following nutrient and food consumptions: sodium and cholesterol, sodium and linoleic acid, cholesterol and linoleic acid, and linoleic acid and processed food. Because these variables are strongly theoretically related, we added error covariance terms between these variables. The goodness-of-fit for the final model was χ^2 (85) = 243.56, $P < .0001$; relative $\chi^2 = 2.87$; CFI = 0.96; RMSEA = .035; and SRMS = .032. The SEM results are depicted in Supplementary Table S5, and for reference, we present the coefficients from the model fitted with variables that were not log-transformed. Similar results were obtained in both SEM analyses. The coefficient of determination (CD) of the model was 0.53.

The bootstrapped test of mediation revealed that the indirect effects of each mediator variable were significant as revealed by their bias-corrected 95% confidence intervals (Figure 3). However, the total indirect effect of age through all mediator variables was not significant [β (95% CI) = -.023 (-.057, .013)]. The direct effect of age on source memory was β = -.595, and thus the proportion of the total effect that was mediated was .037.

Supplementary Table S1. Participants' characteristics and performance on neuropsychological tests by decade. Means and standard deviations appear in parentheses.

Decade	Participants (number)	Sex (women/men)	Education (years)	MMSE	Vocabulary scale (WAIS-R)	BDI
21-30	265	131/134	15.95 (1.87)	29.13 (1.11)	12.99 (1.48)	6.24 (5.01)
31-40	257	128/129	15.93 (3.51)	28.89 (1.15)	12.54 (1.67)	5.93 (5.07)
41-50	256	131/125	14.92 (4.32)	28.70 (1.26)	12.64 (1.76)	6.60 (5.01)
51-60	260	129/131	14.56 (4.77)	28.48 (1.35)	12.70 (1.66)	6.17 (4.90)
61-70	255	131/124	13.58 (4.45)	28.35 (1.35)	12.73 (1.91)	6.49 (4.96)
71-80	264	134/130	12.98 (4.49)	28.02 (1.52	12.87 (1.74)	7.76 (5.09)

Note. MMSE = Mini Mental State Scale, WAIS-R = Wechsler Adult Intelligence Scale-Revised, BDI = Beck Depression Inventory.

Supplementary Table S2. Descriptive statistics; the non-normal variables were log-transformed.

Variables	Mean or number	*SD*	Skewness	Kurtosis
Age (years)	50.12	17.57	-0.08	1.80
Sex (women/men)[a]	784/773			
Education (years)	14.66	4.16	0.38	2.92
Income (ranks)	5.26	1.93	0.27	2.35
Retirement (years)[c]	0.56	1.00	1.51	3.85
Vocabulary scale (WAIS-R)	12.75	1.71	0.09	2.64
Mini Mental State Examination	28.59	1.35	-0.90	3.35
Beck Depression Inventory	6.54	5.03	0.69	2.65
Social Readjustment Rating Scale[c]	2.13	0.59	-0.68	3.37
MIA: Strategy	55.50	11.87	-0.08	2.69
MIA: Task[c]	58.85	6.84	-0.46	3.78
MIA: Capacity	55.67	9.69	-0.26	2.81
MIA: Change	51.20	13.06	-0.08	2.32
MIA: Anxiety	40.90	9.76	0.23	2.50
MIA: Achievement[c]	4.07	0.13	-0.67	3.97
MIA: Locus[c]	3.63	0.15	-0.84	4.38
Illnesses (number)[c]	1.51	0.60	-0.66	3.34
Hypertension (no/yes)[a]	1311/246			
Arrhythmia (no/yes) (no/yes)[a]	1538/19			
Myocardial infarction (no/yes)[a]	1536/21			
Hypotension (no/yes)[a]	1517/40			
Hypercholesterolemia (no/yes)[a]	1480/77			
Presbyopia (no/yes)[a]	1137/420			
Cataracts (no/yes)[a]	1515/42			
Hypoacusis (no/yes)[a]	1524/33			
Diabetes mellitus (no/yes)[a]	1457/100			
Initial insomnia (no/yes)[a]	1436/121			
Terminal insomnia (no/yes)[a]	1507/50			
Intermittent insomnia (no/yes)[a]	1488/69			
Body mass index (k/m2)[c]	3.30	0.15	0.20	3.35
Glucose (mg/dL)[c]	4.53	0.45	-1.61	6.83
Cholesterol (mg/dL)[c]	5.19	0.18	0.39	4.86
Triglycerides (mg/dL)[c]	5.24	0.57	0.06	2.25
Heart rate (bpm)	69.62	11.22	0.21	3.60
Systolic blood pressure (mmHg)[c]	4.77	0.15	0.21	3.07
Diastolic blood pressure (mmHg)[c]	4.31	0.14	0.12	3.52
Mean arterial pressure (mmHg)[c]	4.48	0.14	0.17	3.32
Protein (g)[c]	3.71	0.46	-0.35	3.59
Energy (kcal)[c]	7.71	0.36	-0.21	3.39
Carbohydrates (g)[c]	5.68	0.38	-0.41	3.79
Sucrose (g)[c]	3.69	0.51	-0.18	3.17
Fructose (g)[c]	3.36	0.50	-0.05	3.48

Glucose (g)[c]	3.09	0.52	-0.06	3.46
Calcium (mg)[c]	6.62	0.47	-0.34	3.06
Magnesium (mg)[c]	5.83	0.36	-0.28	3.82
Phosphorus (mg)[c]	7.13	0.38	-0.32	3.64
Potassium (mg)[c]	8.10	0.39	-0.09	3.58
Sodium (mg)[c]	7.48	0.42	-0.09	3.46
Nitrate (g)[c]	4.19	0.58	0.09	3.70
Iron (mg)[c]	2.64	0.35	0.00	3.72
Copper (mg)[c]	1.26	0.42	0.90	4.93
Manganese (mg)[c]	2.70	0.85	-0.04	2.53
Selenium (mcg)[c]	3.59	0.55	-0.09	4.53
Zinc (mg)[c]	2.91	0.48	0.36	4.03
Retinol (UI)[c]	7.80	0.67	0.44	4.03
Carotenes (UI)[c]	8.84	0.68	-0.40	3.35
Alpha-carotene (mcg)[c]	5.71	1.10	-0.67	3.60
Beta-carotene (mcg)[c]	8.01	0.69	-0.48	4.15
Beta-cryptoxanthin (mcg)[c]	6.11	1.11	-1.32	6.46
Lutein & xanthine (mcg)[c]	7.31	0.75	-0.18	3.63
Lycopene (mcg)[c]	8.54	0.79	-1.30	13.77
Thiamine (mg)[c]	0.98	0.25	0.32	3.41
Riboflavin (mg)[c]	1.01	0.27	0.45	3.93
Niacin (mg)[c]	3.02	0.35	0.00	3.35
Pantothenic acid (mg)[c]	2.45	0.59	0.47	3.55
Vitamin B6 glycosylated (mg)[c]	1.83	0.77	0.22	2.80
Folates (mcg)[c]	6.58	0.75	0.21	3.15
Vitamin B12 (mcg)[c]	1.94	0.57	0.49	4.10
Vitamin C (mg)[c]	5.28	0.63	-0.44	3.53
Vitamin D (UI)[c]	5.15	0.66	-0.37	2.94
Alpha-tocopherol (mg)[c]	2.55	0.41	0.28	3.69
Beta-tocopherol (mg)[c]	0.53	0.21	0.73	3.89
Gamma-tocopherol (mg)[c]	2.58	0.54	0.05	3.09
Delta-tocopherol (mg)[c]	1.16	0.42	0.60	3.72
Vitamin K (mcg)[c]	4.41	0.57	0.05	3.65
Alcohol (g)[c]	0.52	0.75	1.57	5.13
Caffeine (g)[c]	3.97	1.74	-0.61	2.45
Cholesterol (g)[c]	5.46	0.56	-0.24	4.16
Butyric acid (g)[c]	0.29	0.19	0.87	4.39
Caproic acid (g)[c]	0.19	0.14	0.91	5.10
Caprylic acid (g)[c]	0.11	0.08	1.38	7.96
Capric acid (g)[c]	0.27	0.16	0.92	4.93
Lauric acid (g)[c]	0.32	0.18	0.73	3.69
Myristic acid (g)[c]	0.94	0.35	0.13	2.54
Palmitic acid (g)[c]	2.56	0.40	-0.01	3.12
Stearic acid (g)[c]	1.80	0.39	0.00	3.18
Palmitoleic acid (g)[c]	0.90	0.27	0.47	3.84
Oleic acid (g)[c]	2.89	0.46	-0.08	3.33
Gadoleic acid (g)[c]	0.14	0.08	1.56	7.11

Linoleic acid (g)[c]	2.22	0.46	0.21	3.12
Alpha-linoleic acid (g)[c]	0.87	0.29	0.85	4.27
Eicosatetraenoic acid (g)[c]	0.07	0.05	2.17	10.74
Eicosapentaenoic acid (g)[c]	0.06	0.06	3.87	35.91
Docosahexaenoic acid (g)[c]	0.16	0.13	2.46	17.70
Canned food (fr)	4.86	1.85	0.36	3.22
Processed food (fr)	3.91	2.60	0.59	2.30
Antidepressants[b c]	0.10	0.44	5.49	36.81
Hypnotics[b c]	0.04	0.26	9.21	101.42
Anxiolytics[b c]	0.08	0.42	6.06	42.19
Hormonal therapy[b]	5.51	20.74	5.30	35.76
Analgesics[b c]	0.13	0.58	5.21	31.21
Amphetamines[b c]	0.03	0.23	9.49	100.56
Cannabis[b c]	0.06	0.36	8.19	79.09
Hallucinogens[b c]	0.00	0.04	33.51	1207.35
Cocaine[b c]	0.02	0.17	14.98	254.85
Tobacco[b c]	2.02	2.24	0.44	1.48
Cigarettes (Number)[c]	0.90	1.05	0.89	2.71
Alcohol[b c]	1.60	1.44	0.74	2.70
Alcohol (gr/week)[c]	2.24	1.83	0.50	2.45
Beer (fr)[c]	0.43	0.76	1.27	2.77
Wine (fr)[c]	0.17	0.52	2.85	9.65
Liqueur (fr)[c]	0.03	0.22	7.06	54.47
Spirit (fr)[c]	0.64	0.81	0.62	1.64
Physical activity[b]	1.29	3.48	4.11	23.62
Television[b]	13.67	9.86	1.71	8.46
Radio[b c]	2.46	1.10	-0.65	2.96
Computer use[b c]	0.63	1.24	1.68	4.23
Reading[b c]	1.73	0.97	-0.18	2.31
Hobbies[b c]	0.74	1.02	1.09	2.90
Cultural activities (fr)	3.77	2.14	-0.49	2.19
Social activities (fr)	4.27	1.85	-0.57	3.00
Source memory (%)	60.25	18.58	-0.23	2.22

Note. [a] Dichotomous variable

[b] Total intake or time = frequency x duration

[c] Log transformed variable

fr = frequency, WAIS-R = Wechsler Adult Intelligence Scale-Revised, MIA = Metamemory in Adulthood Scale

Supplementary Table S3. Linear regressions between each independent variable and correct source memory (%).

Variables	*b*	*SE b*	β	*t*	*P*
Age (years)	-0.617	0.020	-.584	-30.125	.000
sex[a]	-0.387	0.942	-.010	-0.410	.682
Education (years)	0.938	0.114	.210	8.260	.000
Income (ranks)	0.239	0.190	.025	1.260	.208
Retirement (years)[c]	-6.277	0.430	-.338	-14.604	.000
Vocabulary scale (WAIS-R)	0.961	0.277	.088	3.465	.001
Mini Mental State Examination	3.055	0.327	.221	9.330	.000
Beck Depression Inventory	-0.340	0.094	-.092	-3.606	.000
Social Readjustment Rating Scale[c]	7.501	0.762	.237	9.849	.000
MIA: Strategy	-0.039	0.039	-.025	-0.987	.324
MIA: Task[c]	10.283	4.030	.067	2.552	.011
MIA: Capacity	0.351	0.048	.183	7.294	.000
MIA: Change	0.426	0.034	.300	12.618	.000
MIA: Anxiety	-0.386	0.047	-.203	-8.140	.000
MIA: Achievement[c]	-18.115	3.699	-.123	-4.897	.000
MIA: Locus[c]	16.051	3.022	.127	5.312	.000
Illnesses (number)[c]	-3.237	0.764	-.105	-4.236	.000
Hypertension[a]	-10.007	1.165	-.196	-8.589	.000
Arrhythmia[a]	-12.264	3.796	-.072	-3.230	.001
Myocardial infarction[a]	-9.823	4.101	-.061	-2.395	.017
Hypotension[a]	-0.230	2.038	-.002	-0.113	.910
Hypercholesterolemia[a]	-8.250	2.247	-.096	-3.671	.000
Presbyopia[a]	-9.685	0.998	-.231	-9.708	.000
Cataracts[a]	-13.132	2.836	-.115	-4.631	.000
Hypoacusis[a]	-9.368	2.844	-.073	-3.294	.001
Diabetes mellitus[a]	-10.381	1.700	-.137	-6.106	.000
Initial insomnia[a]	2.939	1.674	.042	1.756	.079
Terminal insomnia[a]	-3.401	2.632	-.032	-1.292	.196
Intermittent insomnia[a]	-5.922	2.436	-.066	-2.431	.015
Body mass index (k/m2)[c]	-11.485	3.180	-.091	-3.612	.000
Glucose (mg/dL)[c]	-4.366	1.005	-.106	-4.345	.000
Cholesterol (mg/dL)[c]	-19.376	2.599	-.187	-7.455	.000
Triglycerides (mg/dL)[c]	-4.511	0.793	-.139	-5.688	.000
Heart rate (bpm)	-0.082	0.034	-.050	-2.410	.016
Systolic blood pressure (mmHg)[c]	-32.192	3.028	-.253	-10.632	.000
Diastolic blood pressure (mmHg)[c]	-12.965	3.464	-.095	-3.742	.000
Mean arterial pressure (mmHg)[c]	7.381	3.047	.054	2.422	.016
Protein (g)[c]	3.232	1.032	.080	3.132	.002
Energy (kcal)[c]	4.070	1.321	.078	3.080	.002
Carbohydrates (g)[c]	0.778	1.235	.016	0.630	.529
Sucrose (g)[c]	0.277	0.971	.008	0.286	.775
Fructose (g)[c]	-0.715	0.964	-.019	-0.742	.458

Glucose (g)[c]	0.445	0.937	.012	0.475	.635
Calcium (mg)[c]	0.158	1.021	.004	0.154	.877
Magnesium (mg)[c]	-1.050	1.369	-.020	-0.767	.443
Phosphorus (mg)[c]	2.055	1.256	.042	1.636	.102
Potassium (mg)[c]	-0.941	1.265	-.020	-0.744	.457
Sodium (mg)[c]	2.325	1.125	.052	2.070	.039
Nitrate (g)[c]	-0.492	0.825	-.015	-0.596	.551
Iron (mg)[c]	0.321	1.411	.006	0.227	.820
Copper (mg)[c]	-1.426	1.154	-.032	-1.237	.216
Manganese (mg)[c]	-0.512	0.452	-.023	-1.131	.258
Selenium (mcg)[c]	1.278	0.860	.038	1.487	.137
Zinc (mg)[c]	0.130	1.004	.003	0.129	.897
Retinol (UI)[c]	0.997	0.714	.036	1.396	.163
Carotenes (UI)[c]	-0.362	0.577	-.013	-0.627	.531
Alpha-carotene (mcg)[c]	-0.305	0.353	-.018	-0.865	.387
Beta-carotene (mcg)[c]	-0.056	0.557	-.002	-0.10	.920
Beta-cryptoxanthin (mcg)[c]	-0.541	0.346	-.032	-1.562	.118
Lutein & xanthine (mcg)[c]	-1.150	0.640	-.047	-1.798	.072
Lycopene (mcg)[c]	0.865	0.627	.037	1.381	.168
Thiamine (mg)[c]	3.186	1.965	.042	1.622	.105
Riboflavin (mg)[c]	1.993	1.784	.029	1.117	.264
Niacin (mg)[c]	2.940	1.377	.056	2.135	.033
Pantothenic acid (mg)[c]	-1.234	0.819	-.039	-1.507	.132
Vitamin B6 glycosylated (mg)[c]	1.986	0.594	.082	3.341	.001
Folates (mcg)[c]	-0.429	0.514	-.017	-0.830	.404
Vitamin B12 (mcg)[c]	1.554	0.837	.047	1.857	.064
Vitamin C (mg)[c]	-0.932	0.630	-.032	-1.480	.139
Vitamin D (UI)[c]	-1.133	0.584	-.040	-1.941	.052
Alpha-tocopherol (mg)[c]	4.187	1.144	.091	3.661	.000
Beta-tocopherol (mg)[c]	5.662	2.243	.064	2.524	.012
Gamma-tocopherol (mg)[c]	5.874	0.812	.172	7.231	.000
Delta-tocopherol (mg)[c]	7.681	1.080	.175	7.115	.000
Vitamin K (mcg)[c]	1.299	0.854	.040	1.522	.128
Alcohol (g)[c]	2.850	0.622	.115	4.579	.000
Caffeine (g)[c]	0.344	0.270	.032	1.275	.203
Cholesterol (g)[c]	-1.728	0.681	-.053	-2.538	.011
Butyric acid (g)[c]	1.787	2.492	.018	0.717	.473
Caproic acid (g)[c]	0.700	3.456	.005	0.203	.839
Caprylic acid (g)[c]	2.975	5.672	.013	0.524	.600
Capric acid (g)[c]	4.719	2.903	.041	1.625	.104
Lauric acid (g)[c]	6.301	2.558	.061	2.463	.014
Myristic acid (g)[c]	3.985	1.329	.075	2.997	.003
Palmitic acid (g)[c]	6.479	1.153	.141	5.618	.000
Stearic acid (g)[c]	7.163	1.177	.152	6.086	.000
Palmitoleic acid (g)[c]	8.272	1.744	.120	4.742	.000
Oleic acid (g)[c]	5.036	0.997	.124	5.051	.000
Gadoleic acid (g)[c]	16.002	6.263	.066	2.555	.011

Linoleic acid (g)[c]	7.007	0.991	.172	7.068	.000
Alpha-linoleic acid (g)[c]	6.208	1.598	.098	3.880	.000
Eicosatetraenoic acid (g)[c]	19.040	8.887	.049	2.142	.032
Eicosapentaenoic acid (g)[c]	-1.686	7.806	-.006	-0.216	.829
Docosahexaenoic acid (g)[c]	-0.743	3.853	-.005	-0.193	.847
Canned food (fr)	-1.193	0.260	-.119	-4.586	.000
Processed food (fr)	-1.076	0.180	-.150	-5.981	.000
Antidepressants[b c]	-1.542	1.011	-.037	-1.525	.127
Hypnotics[b c]	-2.722	1.641	-.037	-1.658	.097
Anxiolytics[b c]	-4.046	0.996	-.091	-4.063	.000
Hormonal therapy[b]	0.057	0.021	.064	2.748	.006
Analgesics[b c]	-2.260	0.698	-.070	-3.240	.001
Amphetamines[b c]	-0.624	1.893	-.008	-0.330	.742
Cannabis[b c]	0.268	1.074	.005	0.250	.803
Hallucinogens[b c]	-2.812	6.193	-.006	-0.454	.650
Cocaine[b c]	2.363	2.264	.021	1.044	.297
Tobacco[b c]	-0.456	0.210	-.055	-2.166	.030
Cigarettes (Number)[c]	-0.209	0.447	-.012	-0.468	.640
Alcohol[b c]	-1.199	0.318	-.093	-3.774	.000
Alcohol (gr/week)[c]	0.342	0.258	.034	1.330	.184
Beer (fr)[c]	4.666	0.566	.191	8.240	.000
Wine (fr)[c]	2.283	0.699	.063	3.264	.001
Liqueur (fr)[c]	-2.238	2.305	-.027	-0.971	.332
Spirit (fr)[c]	-2.229	0.592	-.097	-3.767	.000
Physical activity[b]	0.752	0.110	.141	6.848	.000
Television[b]	-0.102	0.049	-.054	-2.095	.036
Radio[b c]	1.229	0.425	.073	2.893	.004
Computer use[b c]	3.690	0.332	.247	11.100	.000
Reading[b c]	1.487	0.466	.078	3.194	.001
Hobbies[b c]	1.100	0.390	.060	2.820	.005
Cultural activities (fr)	1.322	0.225	.152	5.886	.000
Social activities (fr)	0.778	0.258	.077	3.017	.003

Note. [a] Dichotomous variable: sex (female = 1, male = 2); diseases (no = 0, yes = 1)
[b] Total intake or time = frequency x duration
[c] Log transformed variable
fr = frequency, WAIS-R = Wechsler Adult Intelligence Scale-Revised, MIA = Metamemory in Adulthood Scale

Supplementary Table S4. Correlations among variables and descriptive statistics.

Variables	1	2	3	4	5	6	7	8	9	10	11	12	13	14	15	16
1. Age (years)	—															
2. MMSE	-.27***	—														
3. MIA: Anxiety	.23***	-.16***	—													
4. Heart rate (bpm)	-.05*	.03	-.03	—												
5. MAP (mmHg)[b]	.38***	-.13***	.00	.18***	—											
6. Sodium (mg)[b]	-.13***	.00	.01	.00	-.04	—										
7. Cholesterol (g)[b]	-.20***	.00	-.04	.07**	.01	.54***	—									
8. Linoleic acid (g)[b]	-.26***	.05*	-.01	.04	-.09***	.77***	.51***	—								
9. Processed food (fr)	.15***	.02	-.02	.00	.07**	-.06*	-.08**	-.16***	—							
10. Wine (fr)[b]	.09***	-.03	-.03	-.01	.04	.01	-.02	-.02	-.03	—						
11. Anxiolytics[a b]	.09***	-.01	.04	.00	.00	-.01	-.03	-.03	-.02	.07**	—					
12. Hormonal therapy[a]	.09***	-.01	.02	.00	-.04	-.04	-.03	-.01	.01	.05*	.10***	—				
13. Physical activity[a]	-.15***	.02	-.07**	-.07**	-.01	.10***	.04	.09***	-.01	-.01	-.01	-.04	—			
14. Computer use[a b]	-.29***	.12***	-.18***	.08**	-.08**	.09***	.10***	.12***	-.01	.00	-.07**	-.07**	.14***	—		
15. Hobbies[a b]	.21***	-.07**	.03	-.03	.05*	.04	-.01	.02	.00	.04	.01	.07**	-.05*	-.08**	—	
16. Source memory (%)	-.58***	.22***	-.20***	-.05*	.05*	.05*	-.05*	.17***	-.15***	.06*	-.09***	.06*	.14***	.25***	.06*	—
Mean	50.1	28.6	40.9	69.6	87.7	1929.2	275.0	9.3	3.9	0.5	0.4	5.5	1.3	5.0	3.0	60.3
SD	17.6	1.4	9.8	11.2	12.2	849.3	170.7	5.2	2.6	1.5	2.9	20.7	3.5	12.8	6.2	18.6
Mean (log)					4.5	7.5	5.5	2.2		0.2	0.1			0.6	0.7	
SD (log)					0.1	0.4	0.6	0.5		0.5	0.4			1.2	1.0	

Note. [a] Total intake or time = frequency x duration

[b] Log transformed variable

MMSE = Mini Mental State Scale, MIA = Metamemory in Adulthood Scale, MAP = mean arterial pressure, fr = frequency

* p < 0.05, ** p < 0.01, *** p < 0.001

Supplementary Table S5. Structural equation modeling results. Coefficients in the first column are from the model without log-transformed variables. The remaining results correspond to the model with log-transformed variables. Significant results were identical in both models.

		b	*b* log	β	*SE* β	*P*
Age (years) ⟶	MMSE	-0.021	-0.021	-.272	.023	.000
	MIA: Anxiety	0.125	0.125	.225	.024	.000
	Heart rate (bpm)	-0.032	-0.032	-.050	.025	.046
	MAP (mmHg)[b]	0.260	0.003	.381	.021	.000
	Sodium (mg)[b]	-6.674	-0.003	-.131	.025	.000
	Cholesterol (g)[b]	-1.671	-0.006	-.196	.024	.000
	Linoleic acid (g)[b]	-0.078	-0.007	-.257	.023	.000
	Processed food (fr)	0.022	0.022	.147	.025	.000
	Wine (fr)[b]	0.008	0.003	.085	.025	.001
	Anxiolytics[a b]	0.011	0.002	.085	.025	.001
	Hormonal therapy[a]	0.101	0.101	.087	.025	.001
	Physical activity[a]	-0.030	-0.030	-.149	.025	.000
	Computer use[a b]	-0.178	-0.021	-.293	.023	.000
	Hobbies[a b]	0.071	0.012	.215	.024	.000
MMSE ⟶	Source memory (%)	0.880	0.873	.063	.021	.002
MIA: Anxiety		-0.111	-0.103	-.054	.021	.009
Heart rate (bpm)		-0.109	-0.107	-.065	.020	.002
MAP (mmHg)[b]		0.110	10.090	.074	.022	.001
Sodium (mg)[b]		-0.002	-3.361	-.076	.033	.022
Cholesterol (g)[b]		-0.005	-2.237	-.068	.024	.005
Linoleic acid (g)[b]		0.372	4.148	.101	.033	.002
Processed food (fr)		-0.480	-0.489	-.068	.020	.001
Wine (fr)[b]		0.615	1.943	-.047	.020	.007
Anxiolytics[a b]		-0.333	-2.085	.068	.020	.019
Hormonal therapy[a]		0.061	0.061	.054	.020	.001
Physical activity[a]		0.257	0.231	.043	.020	.034
Computer use[a b]		0.083	1.140	.076	.021	.000
Hobbies[a b]		0.202	1.016	.056	.020	.006
Age (years)		-0.598	-0.595	-.563	.022	.000

Note. [a] Total intake or time = frequency x duration
[b] Log transformed variable
MMSE = Mini Mental State Scale, MIA = Metamemory in Adulthood Scale, MAP = mean arterial pressure, fr = frequency
